# Genome sequence of *Vibrio harveyi* strain TUMSAT-2019, isolated from kuruma shrimp, *Penaeus japonicus*

**DOI:** 10.1128/mra.00746-24

**Published:** 2024-12-20

**Authors:** Hajime Yuasa, Keiichiro Koiwai, Kayo Konishi, Miho Furukawa, Reiko Nozaki, Hidehiro Kondo, Ikuo Hirono

**Affiliations:** 1 Laboratory of Genome Science, Tokyo University of Marine Science and Technology, Tokyo, Japan; Montana State University, Bozeman, Montana, USA

**Keywords:** vibriosis, aquaculture, shrimp, whole-genome sequencing

## Abstract

The Gram-negative bacterium *Vibrio harveyi* is a serious shrimp pathogen. Here, we present the genome sequence of *Vibrio harveyi* TUMSAT-2019, which was isolated from a kuruma shrimp (*Penaeus japonicus*) that originated from a shrimp farm in Okinawa Prefecture. The assembly totaled 5.7 Mbp, consisting of two chromosomes with no plasmid.

## ANNOUNCEMENT

The Gram-negative bacterium *Vibrio harveyi* is ubiquitous in aquatic systems (1) and is known as a bacterial pathogen of marine fish and invertebrates, including shrimp, in aquaculture (2). In shrimp aquaculture, *V. harveyi* is responsible for various cases, for example, luminous vibriosis (3), eye lesions (4), and acute hepatopancreatic necrosis disease (AHPND) (5). We isolated *V. harveyi* strain TUMSAT-2019 from a kuruma shrimp (*Penaeus japonicus*) that originated from a shrimp farm on Kumejima Island, Okinawa Prefecture, Japan, in 2019. We streaked the hepatopancreas of a shrimp onto a Bacto Heart Infusion (HI) agar supplemented with 2.5% (wt/vol) NaCl, and the obtained single colony was re-streaked onto a new HI agar supplemented with 2.5% (wt/vol) NaCl. 16S rDNA sequence analysis for a single colony indicated that it is related to *V. harveyi*.

Here, we present the genome sequence of *V. harveyi* TUMSAT-2019. We performed Oxford Nanopore long-read sequencing to obtain a draft assembly and performed the DNBSEQ short-read sequencing to polish the draft assembly. For both preparations, the strain was cultured with Luria-Bertani medium supplemented with 2.0% (wt/vol) NaCl. An aliquot (4 mL) of expanded culture was concentrated (5,000 × *g*) and genomic DNA was extracted using a NucleoBond AXG 20 column (Macherey-Nagel) and the NucleoBond buffer set III (Macherey-Nagel). The obtained genomic DNA was dissolved in 1/10 low Tris-EDTA (TE) buffer and then qualified by Qubit (Invitrogen) prior to sequencing. No shearing or size selection of the extracted DNA was performed before library preparation.

For long-read sequencing, a DNA library was prepared using the Rapid Barcoding Sequencing Kit (SQK-RBK004, Oxford Nanopore Technologies) and was sequenced using an R9.4.1 flow cell on a GridION platform. Sequenced data were subjected to base calling using Guppy v.7.1.4 (6), which generated 122,628 reads (688.6 Mb; Read length N_50_, 8,177 bp). For short-read sequencing, a DNA library for DNBSEQ sequencing was prepared using the MGIEasy PCR-Free DNA Library Prep Set (MGI Tech), and 2 × 150 bp paired-end sequencing was performed using the DNBSEQ-T7 (MGI Tech), yielding 2,655,200 paired-end read. The raw sequencing data were subjected to quality trimming using Ktrim v.1.5.1 (7) to filter out adapter sequences and low-quality data.

Hybrid assemblies combining long and short reads were performed using the hybracter pipeline v.0.7.3 (8). In this tool, long reads are filtered with Filtlong v.0.2.1 (9) and adapters trimmed with Porechop ABI v.0.5.0 (10). Quality control of short reads is performed with fastp v.0.23.4 (11). Long reads were assembled and circularized with Flye v.2.9.3 (12) and polished with Medaka v.1.8.0 (13). The chromosomes were polished using short reads with Polypolish v.0.6.0 (14) and Pypolca v.0.3.1 (15), then chromosomes were reoriented manually to begin with the dnaA gene for the chromosome 1 and the parA gene for the chromosome 2, respectively.

The assembled genome consisted of two contigs representing two chromosomes (3,528,099 bp; 110× coverage and 2,255,452 bp; 93× coverage), totaling 5,783,551 bp, with the overall GC content of 44.9%. Annotation that used DFAST v.1.2.0 (16) showed the genome sequence contains 5,150 protein-coding genes, 36 rRNA genes, and 134 tRNA genes (Table 1). The completeness of the genome was 99.54% using DFAST_QC (17).

**TABLE 1:** Assembly statistics of the *V. harveyi* TUMSAT-2019 genome

Contig	GenBank Accession no.	Topology	Length (bp)	GC content (%)	No. of :		
					CDS	rRNAs	tRNAs
Chromosome 1	AP031614	Circular	3,528,099	45.1	3,114	33	118
Chromosome 2	AP031615	Circular	2,255,452	44.9	2,036	3	16

## Data Availability

The *V. harveyi* TUMSAT-2019 genome is available in DDBJ/EMBL/GenBank under the accession numbers AP031614 to AP031615. The raw read data are also available with the accession numbers DRR570219 to DRR570220.
